# A parallel metaheuristic for large mixed-integer dynamic optimization problems, with applications in computational biology

**DOI:** 10.1371/journal.pone.0182186

**Published:** 2017-08-15

**Authors:** David R. Penas, David Henriques, Patricia González, Ramón Doallo, Julio Saez-Rodriguez, Julio R. Banga

**Affiliations:** 1 BioProcess Engineering Group. IIM-CSIC, Spanish National Research Council. C/Eduardo Cabello, 6. E-36208 Vigo, Spain; 2 Computer Architecture Group. Department of Electronics and Systems. University of A Coruña. Campus de Elviña s/n. 15071 A Coruña, Spain; 3 RWTH Aachen University, Faculty of Medicine, Joint Research Centre for Computational Biomedicine, Aachen D-52074, Germany; 4 European Molecular Biology Laboratory, European Bioinformatics Institute (EMBL-EBI), Hinxton, CD10 1SD, United Kingdom; Universitat Pompeu Fabra, SPAIN

## Abstract

**Background:**

We consider a general class of global optimization problems dealing with nonlinear dynamic models. Although this class is relevant to many areas of science and engineering, here we are interested in applying this framework to the reverse engineering problem in computational systems biology, which yields very large mixed-integer dynamic optimization (MIDO) problems. In particular, we consider the framework of logic-based ordinary differential equations (ODEs).

**Methods:**

We present saCeSS2, a parallel method for the solution of this class of problems. This method is based on an parallel cooperative scatter search metaheuristic, with new mechanisms of self-adaptation and specific extensions to handle large mixed-integer problems. We have paid special attention to the avoidance of convergence stagnation using adaptive cooperation strategies tailored to this class of problems.

**Results:**

We illustrate its performance with a set of three very challenging case studies from the domain of dynamic modelling of cell signaling. The simpler case study considers a synthetic signaling pathway and has 84 continuous and 34 binary decision variables. A second case study considers the dynamic modeling of signaling in liver cancer using high-throughput data, and has 135 continuous and 109 binaries decision variables. The third case study is an extremely difficult problem related with breast cancer, involving 690 continuous and 138 binary decision variables. We report computational results obtained in different infrastructures, including a local cluster, a large supercomputer and a public cloud platform. Interestingly, the results show how the cooperation of individual parallel searches modifies the systemic properties of the sequential algorithm, achieving superlinear speedups compared to an individual search (e.g. speedups of 15 with 10 cores), and significantly improving (above a 60%) the performance with respect to a non-cooperative parallel scheme. The scalability of the method is also good (tests were performed using up to 300 cores).

**Conclusions:**

These results demonstrate that saCeSS2 can be used to successfully reverse engineer large dynamic models of complex biological pathways. Further, these results open up new possibilities for other MIDO-based large-scale applications in the life sciences such as metabolic engineering, synthetic biology, drug scheduling.

## Introduction

Global optimization is being increasingly used in engineering and across most basic and applied sciences [1–4], including areas from life sciences such as bioinformatics and computational systems biology [5–9]. In the case of chemical and biological processes, during the last decade there has been a growing interest in modelling their dynamics [10], i.e. developing kinetic models which are able to encapsulate the time-varying nature of these systems. As a consequence, many research efforts are now being invested in exploiting those dynamic models by mathematical optimization techniques. These formulations belong to the class of dynamic optimization problems (or open loop optimal control). The most general formulation is that of mixed-integer dynamic optimization (MIDO), where part of decision variables are discrete (binary or integer) [11].

Although many dynamic optimization problems consider how to extract useful operating policies and/or designs from a dynamic model, such formulations can also be applied to the model building process itself, i.e. to the so-called reverse engineering problem [12–19], which is known to be extremely hard [10].

Here we consider this general problem of reverse engineering in computational biology by means of mixed-integer nonlinear dynamic optimization (MIDO). Our goal is to be able to handle large-scale nonlinear kinetic models, so we focus on the solution of this class by means of suitable global optimization methods. Broadly speaking, MIDO problems can be solved using deterministic or stochastic global optimization methods. In the case of deterministic methods, many advances have been made in recent years (see [11, 20–22] and references therein). Although these deterministic methods can guarantee global optimality in some cases, unfortunately they suffer from lack of scalability, i.e. the associated computational effort increases very rapidly with problem size.

Alternatively, although stochastic algorithms for global optimization cannot offer guarantees of global optimality, they usually reach a region near to the global solution in acceptable execution time, at least for small and medium scale problems. However, for larger problems the computational cost of purely stochastic methods can be very large [5, 23]. Several hybrid approaches [24–28] have tried to benefit from the best of both approaches by combining global stochastic methods with efficient (local) deterministic optimization methods. In this context, metaheuristics (i.e. guided heuristics) have been particularly successful, ensuring the proper solution of these problems by adopting a global stochastic optimization approach, while keeping the computational effort under reasonable values thanks to efficient local optimization solvers [29, 30].

Here we consider the general MIDO problem as described above. We present a new parallel method based on extensions of an enhanced scatter search metaheuristic [30, 31] which has shown good performance in simpler non-linear programming and optimal control problems. We formulate the reverse engineering problem as a MIDO using the framework of logic-based ordinary differential equations (ODEs) [32, 33]. In this framework, the logic components need to be estimated along with the continuous parameters in order to describe the dynamic behavior defined by the structure of the system of ODEs. The resulting problem is very hard due to its multimodal, non-linear and highly constrained nature.

Our contribution resides at the interface between computational systems biology and computer science (high performance computing), with an emphasis on using the latter to help the former. The merits of the logic-based ODEs framework have been illustrated previously [32, 33]. But, as already recognized in Henriques et al [33], more work was needed regarding the computational efficiency of the optimization methods. This has been precisely our main objective here: how to improve the robustness and efficiency of the numerical solution of these problems by developing a better algorithm and its corresponding high-performance computing implementation.

In order to be able to handle these more complex MIDO problems in realistic times, we have focused on developing a parallel cooperative strategy that scales up well with problem size. Parallel strategies for metaheuristics have been a very active research area during the last decade [34, 35]. In the area of computational biology, parallel methods have already shown promising results in non-linear optimization problems ([36, 37]). For the case of mixed-integer nonlinear programming, a few researchers have considered the development of parallel methods [38–40], but there is a lack of studies in the case of the mixed-integer nonlinear dynamic optimization problems considered here.

The aim of this paper is to explore this direction further considering extensions of a recent parallel self-adaptive implementation of the enhanced Scatter Search algorithm [41] so it can handle general MIDO problems of realistic size. It should be noted that there are several key differences and important novel aspects with respect to our previous work:

- we extend and generalize the formulation considered in Henriques et al [33] by adopting a generic mixed-integer optimal control approach, without using relaxations and/or transformations of the original problem during the solution strategy
- we present a new solution strategy based on a parallel cooperative optimization method with specific extensions to handle large mixed-integer problems, and new mechanisms of self-adaptation tailored to this class of problems
- we illustrate the performance of our approach by considering a set of very challenging case studies, obtained in different high-performance computing infrastructures, including traditional parallel machines and a public cloud-computing platform. In particular, we show how our new method can successfully exploit cloud computing resources

The organization of this paper is as follows. In the Background section we present the general mixed-integer dynamic optimization formulation and we outline the solution strategy, which is based on the control vector parameterization direct method. This solution approach transforms the problem into a master mixed-integer nonlinear programming problem with an inner initial value problem. We solve the outer problem by means of an improved parallel metaheuristic, which is described in the following section. We then evaluate this approach considering several challenging reverse engineering case studies. We evaluate the performance of the proposal using these cases on a local cluster, a large supercomputer and in the cloud (using Microsoft Azure), demonstrating its good efficiency and scalability, as discussed in the Results and Discussion Section. Finally, in the Conclusions Section we summarize the main contributions of this work.

## Background

In this section we present the general statement of the class of problems considered. Next, we describe the numerical approach used to solve it, based on the so-called control vector parameterization direct method. We then describe the background regarding the scatter search metaheuristic which will used as the basis for the parallel method presented in the following section.

### Mixed integer dynamic optimization problem

The general mixed-integer dynamic optimization problem (MIDO), also called mixed-integer optimal control (MIOC) problem [21], is usually formulated as finding the set of discrete (integer or binary), time-dependent (stimuli or controls) and time-independent parameters, to optimize (minimize or maximize) a pre-defined cost function (which in optimal control is generically called performance index), while satisfying a set of dynamic and algebraic constraints. In mathematical form, it is usually formulated as follows:

Find **u**(*t*), **i**(*t*), **p** and *t*_f_ so as to minimize (or maximize):

$$\begin{array}{c} J=G_{{\text{t}}_{\text{f}}}\left(x,u,i,p,t_{\text{f}}\right)+\int_{t_{0}}^{t_{\text{f}}}F\left(x\left(t\right),u\left(t\right),i\left(t\right),p,t\right)\text{d}t \end{array}$$ (1)

subject to:

$$\begin{array}{ccc} f(\dot{x}\left(t\right),x\left(t\right),u\left(t\right),i\left(t\right),p,t)=0, & \; & x(t_{0})=x_{0} \end{array}$$ (2)

$$\begin{array}{ccc} g(x(t),u(t),i(t),p,t)\leq 0, & \; & l=\bar{1,m_{\text{e}}+m_{\text{i}}} \end{array}$$ (3)

$$\begin{array}{c} u_{\text{L}}\leq u(t)\leq u_{\text{U}}, \end{array}$$ (4)

$$\begin{array}{c} i_{\text{L}}\leq i(t)\leq i_{\text{U}}, \end{array}$$ (5)

$$\begin{array}{c} p_{\text{L}}\leq p\leq p_{\text{U}}, \end{array}$$ (6)

where **x**(*t*) ∈ *X* ⊆ R^*n*_x_^ is the vector of state variables, **u**(*t*) ∈ *U* ⊆ R^*n*_u_^ is the vector of real valued control variables, **i**(*t*) ∈ *I* ∈ Z^*n*_i_^ is the vector of integer control variables, **p** ∈ *P* ⊆ R^*n*_p_^ is the vector of time-independent parameters, *t*_f_ is the final time of the process, *m*_e_, *m*_i_ represent the number of equality and inequality constraints, **f** is the set ordinary differential equations describing the dynamics of the system (plus the corresponding initial conditions), **g** is the set of state constraints (path, pointwise and final time constraints), and **u**_L_, **i**_L_, **p**_L_, **u**_U_, **i**_U_, **p**_U_ correspond to the lower and upper bounds for the control variables and the time-independent parameters. In the formulation above, known as the general Bolza problem, *G*_t_f__ is a terminal cost function, and *F* is an integral cost function.

The MIDO formulation above can be used to solve problems from widely different areas, including aeronautics, chemical engineering, mechanical engineering, transport, medicine, systems biology, synthetic biology and industrial biotechnology [11, 20, 33, 42–50]. In the particular context of reverse engineering complex biological networks [33], our aim is to use the above framework to simultaneously identify the underlying network topology, its regulatory structure, the time-dependent controls (e.g. stimuli) and time-invariant model parameters, consistent with existing experimental data (time-series). An alternative would be to carry out parameter estimation based on real-values for each individual and possible model structure, but this option becomes prohibitively expensive for any realistic case.

### Solution strategy

Methods for the numerical solution of dynamic optimization (optimal control) problems can be broadly classified under three categories: dynamic programming, indirect and direct approaches. Dynamic programming [51, 52] suffers form the so called *curse of dimensionality*, so the latter two are the most promising strategies for realistic problems. Indirect approaches were historically the first developed, and are based on the transformation of the original optimal control problem into a multi-point boundary value problem using Pontryagin’s necessary conditions [53, 54]. Direct methods are based on discretization of the control (sequential strategy [55]), or both the control and the states (simultaneous strategy [56]).

Here, our strategy uses a direct approach and consists of a first (transformation) step, (transcribing the original MIDO problem into a mixed-integer nonlinear programming problem, MINLP), followed by a second (numerical solution) step (the actual solution of the MINLP by the novel cooperative scatter search metaheuristic). We have chosen the control parameterization approach [11], that consists in discretizing the control variables (**u**(*t*) and **i**(*t*)) into a number of elements, and then approximating the controls in each element by means of certain basis functions. The control variables are, thus, parameterized using **w**_**u**_ ∈ R^*ρ*^ and **w**_**i**_ ∈ Z^*ρ*^, which become time-invariant decision variables. With this approach, the original problem is transformed from an infinite dimensional problem into a finite dimension mixed-integer non-linear programming outer problem, that can be solved using a suitable MINLP solver. Note that, as a consequence, the evaluation of the objective function and constraints requires the solution of an inner problem (the system dynamics), by a suitable initial value problem (IVP) solver.

In summary, our strategy results in a numerical optimization problem composed of:

- an outer mixed-integer nonlinear programming (MINLP) problem: due its non-convexity, we need to use global optimization methods, as already mentioned in the introduction. Based on our previous experiences with different stochastic global methods and their hybrids with local solvers [24, 26, 30, 31, 41, 57, 58], here we decided to extend a metaheuristic based on scatter search, combining it with a efficient local MINLP solver [59], as described below.
- an inner initial value problem (IVP), i.e. the nonlinear dynamics that need to be integrated for each evaluation of the cost functional and constraints. Here we solve the IVP using the state-of-the-art solvers for numerical integration of differential equations included in the SUNDIALS package [60]. It should be noted that local optimization methods to be used require the numerical computation of gradients of the objective and/or constraints with respect to the decision variables. If this is the case, an efficient procedure is to use first order parametric sensitivities to compute such information [61]. The sensitivity equations can be obtained by a chain rule differentiation of the system defined in Eq 2 with respect to the decision variables. They can be efficiently solved in combination with the original system. Here we have used SUNDIALS [60], which includes CVODES, an efficient sensitivity solver.

We now proceed to detail the parallel metaheuristic method developed for solving this problem.

### Scatter search and recent extensions

Scatter Search (SS) [62] is one of the most popular metaheuristics to solve global optimization problems. It can be regarded as a population-based algorithm that creates new solutions through iterative steps of diversification, improvement, combination and population update. Compared to other metaheuristics, SS uses a low number of population members.

**Fig 1:**
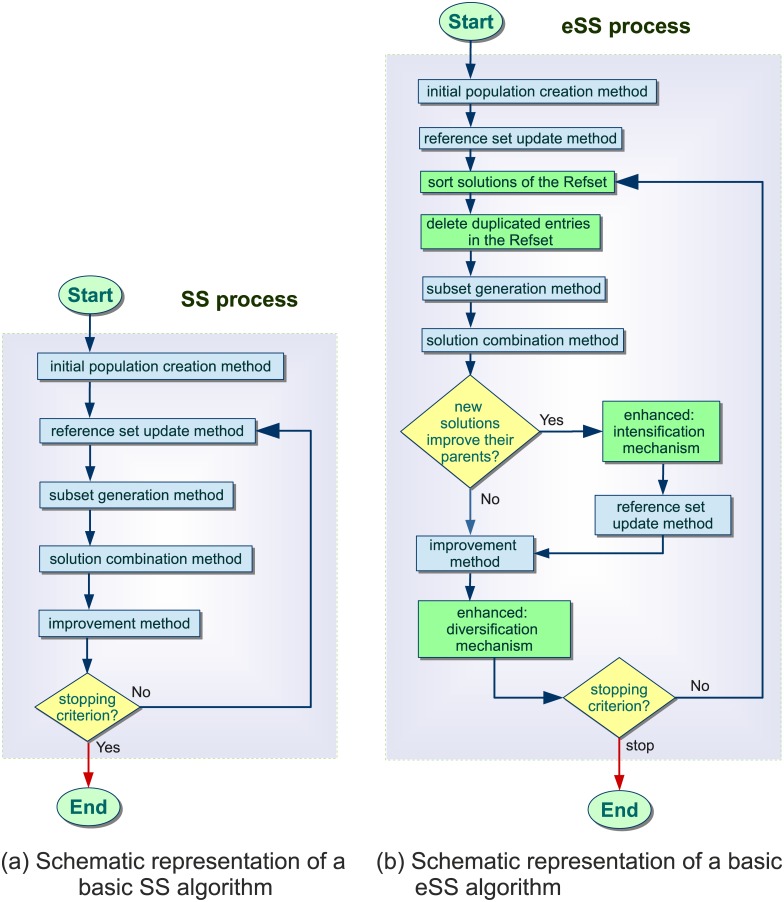
Schematic representation of sequential algorithms.

The SS strategy involves 5 steps, illustrated in Fig 1(a): (1) SS begins by producing an initial population of solutions within the search space; (2) the initial *Reference Set* is then created with a set of representative solutions of the population; (3) a generation method selects a subset of solutions from the reference set; (4) the solutions in this subset are combined to obtain new solutions; (5) finally, an improvement procedure is applied to the previous solutions. The update method (step 2) creates again the reference set for the next iteration, and this procedure is repeated until the end of the search.

A recent implementation of this procedure, named *enhanced Scatter Search* (eSS) [30, 31], presents a straightforward yet effective design that helps to beat well known issues in nonlinear dynamic systems optimization such as flat areas, noise, nonsmoothness, and/or discontinuities. Fig 1(b) graphically shows the main functionalities of the eSS. Novel mechanisms, highlighted in green in the figure, are included to achieve a good trade-off between intensification (local search) and diversification (global search):

- A rather small population size is used, even for large-scale problems. However, more search directions are allowed than in the original SS by means of a new combination scheme. The diversity in the search is preserved while the number of evaluations required is not increased.
- A new intensification mechanism in the global phase exploits the promising directions defined by a pair of solutions in the reference set.
- A heuristic local search method accelerates the convergence, specially for large-scale problems.
- It makes use of memory to infer whether a solution become stagnant in a local optimum or whether it is alongside of already visited solutions.

Despite the success of the strategies included in the eSS, for large-scale problems involving dynamic systems, it still requires significant computation times. Besides, the eSS method needs the tuning of a number of configuration settings that may have a great impact in the algorithm performance, thus requiring a number of initial exploratory runs and, therefore, further increasing the computation times. With the aim of solving these issues, we recently developed a parallel method named self-adaptive Cooperative enhanced Scatter Search (saCeSS) [41] and demonstrated its advantages for the solution of hard parameter estimation problems involving nonlinear dynamic models. Essentially, the saCeSS method is a novel parallel metaheuristic that follows an island-model strategy where a set of independent eSS threads (islands) exchange information (solutions and settings) among them to improve the convergence through cooperation, effectively implementing a self-tuning mechanism of the algorithm. Several key functionalities have been included in saCeSS in order to overcome the limitations of eSS:

- a coarse-grained parallelization following a master-slave model, where the master manages the control of the cooperation between slaves (islands), since an excessive of cooperation results in adverse impacts on diversity
- an exchange of information handled taking into account the quality of the solutions obtained by each individual process, as an alternative to time elapsed, to achieve more effective cooperation between processes
- an asynchronous communication protocol to tackle the exchange of information between processes, avoiding inactive processes when they are waiting for information exchanged from other processes
- a self-adaptive mechanism in the master process that performs a scoreboard used to dynamically tune the settings of the islands based on their individual progress

## A parallel cooperative scatter search for mixed integer optimization

Since the saCeSS method described above has demonstrated its potential for solving very challenging non-linear programming (NLP) problems, there was an interest in extending this method so it can be applied to large mixed-integer nonlinear programming and mixed-integer dynamic optimization problems. As a result, we present here a new method, saCeSS2, resulting from modifications and extensions of the original saCeSS method along three main directions:

1. addition of an efficient local solver for mixed-integer optimization problems
2. changes to the self-adaption mechanisms in order to avoid premature stagnation of the convergence
3. addition of new mechanisms to ensure diversity while keeping parallel cooperation

Regarding the local solver, we have incorporated a trust region sequential quadratic programming solver, called *Mixed-Integer Sequential Quadratic Programming* (MISQP) [59, 63]. It assumes that the model functions are smooth: an increment of a binary or an integer variable can produce a small change of function values, though it does not require the mixed-integer function to be convex or relaxable, i.e. the cost function is evaluated only with discrete values in the integer or boolean parameters.

The preliminary tests applying the previous saCeSS algorithm to mixed-integer problems using the MISQP local solver brought to light a problem of premature convergence due to a quick lose of diversity in the islands. Although both eSS and saCeSS algorithms include their own mechanisms to maintain the desired diversity during the algorithm progress, we observed that in mixed-integer problems a promising incoming solution in an island acted as an attractor for the members of the *RefSet*, bringing them fast to the vicinity of this new value. Thus, we introduced two new strategies in the saCeSS2 method to allow for a dynamic breakout from local optima, and to further preserve the diversity in the search for these problems, avoiding prematurely stagnation:

- first, we needed to avoid the premature convergence observed in MINLP problems (cooperation between islands decreases too fast as the algorithm converges, since many of them stagnate). Thus, the criteria used in the original saCeSS method to trigger the reconfiguration (tuning) of those islands that are not progressing in the search should be accommodated for MINLP problems, relaxing the adaptive conditions to allow for an earlier escape from the stagnated regions.
- Second, we observed that in mixed-integer problems, when an island stagnates, most of the times is due to the lost of diversity in the *RefSet*. Thus, we decided to further promote diversity by a modified strategy: once an island requests a reconfiguration, most of the members of the *RefSet*, except for two solutions, are randomly initialized again.

**Fig 2:**
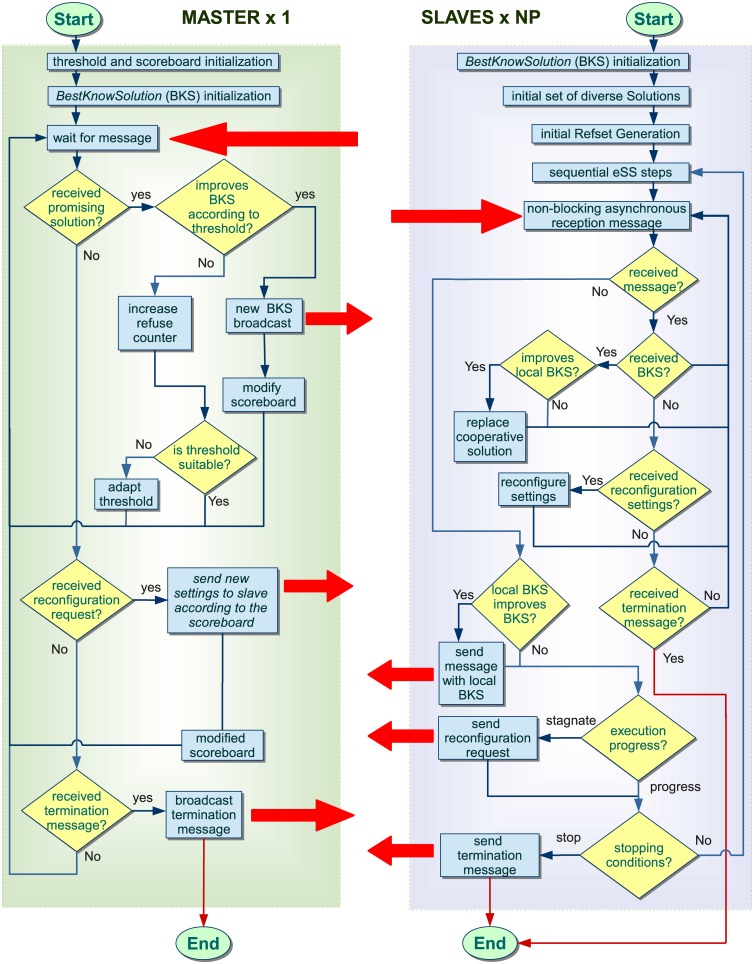
Schematic representation of saCeSS2 algorithm.

Fig 2 summarizes the new saCeSS2 method for MINLP/MIDO problems. The master process is in charge of the control of the cooperation and the scoreboard for the islands’ tuning. At the beginning of the algorithm, both at master and at slaves, a local variable *BestKnownSol* is set to monitor the best solution shared in the cooperation among slaves. The master process also sets the initial communication threshold *ϵ* and initiates the scoreboard to monitor the progress of each slave. Then, a loop is carried out until a stopping criteria is reached, where the master waits for the messages coming from the slaves. In the cooperation stage the master manages the appearance of good solutions received from slaves. Then, with the aim of controlling the cooperation between slaves, only when the incoming candidate solution significantly improves the current *BestKnownSol*, this variable is updated and broadcasted. The master process is able to self-tuning the cooperation threshold based on the number of incoming solutions that are refused with the current criterion. Besides, when a new incoming solution deserves to become a cooperative solution spread to the rest of the slaves, there is an increment on the score of the slave that achieved that solution. The master process also manages the slaves adaptation requests. To accurately identify those islands that are not progressing in the search, the master process would need additional information from slaves. The solution implemented is that each slave resolves whether it has stagnated or not. If promising cooperative solutions are arriving from the master but the island cannot improve its local best known solution, it will ask the master for a reconfiguration. Then, the master will communicate to that island one of the configuration settings of the islands on the top of the scoreboard. Finally, if the master receives a termination message from one of the slaves, it broadcast the termination request to the rest.

The slaves perform the classic steps of the sequential eSS. Additionally new steps are included to implement cooperation and self-tuning. First, a reception memory buffer retains the messages arriving from the master that have not been processed yet, thus, the communications are all done in a non-blocking asynchronous way. The slave inspects its reception memory buffer looking for new best solutions from the master. When new solutions have arrived, the slave checks whether the new solutions improve the local *BestKnownSol* or not. If a new solution improves the local one, this new solution upgrades to *BestKnownSol*. Then, the slave also checks the reception of new reconfiguration settings. Note that, as already explained, all the communications between slaves and master are asynchronous, thus, the request for a reconfiguration is also a non-blocking operation. This means that the slave goes on with its execution until the message with the reconfiguration settings arrive. Besides, in the reception step, the slave also checks the arrival of termination messages from the master. If a termination message arrives, the slave finishes its execution.

After the reception step, the slave checks if its best local solution improves in, at least, an *ϵ* the *BestKnownSol*. If so, *BestKnownSol* is updated with the best local solution and the slave sends the promising result to the master. The *ϵ* used in the slaves is different from the *ϵ* used in the master process. The slaves use a smaller *ϵ* so that many *promising* solutions are sent to the master. The master has to make a decision on which of those incoming solutions should be spread to the rest of the slaves. This decision is based on the quality of the incoming solutions. Thus, the *ϵ* used by the master begins with high value and decreases as long as the number of refused solutions get larger and no incoming solution overcomes the current *ϵ*.

To conclude the iteration, an adaptive step is accomplished. Each slave decides if it is progressing in the search based on:

- *Number of evaluations performed since its last cooperation*:
  $$\begin{array}{c} N_{\text{eval}}>N_{\text{par}}\times 500 \end{array}$$
  where *N*_eval_ is the number of evaluations performed by this process since its last cooperation with the master and *N*_par_ is the number of parameters of the problem.
- *Balance between the received and sent solutions*:
  $$\begin{array}{c} recvSolutions>(4\times sendSolutions)+10 \end{array}$$
  adaptation is requested when the number of received solutions is significantly greater than the number of solutions sent (with a minimum value of 10, to avoid requests at the beginning of the process), that is, if other slaves are cooperating much more than itself.

In summary, if a process recognizes that it has stagnated, it sends a request for reconfiguration to the master process. In response to these requests, the master sends to those slaves the most encouraging settings, i.e., those that are on the top of the scoreboard. In order to inject further diversity into those reconfigured islands, most of the members of their *RefSet* are randomly re-initialized.

The saCeSS2 algorithm repeats the external loop until the stopping criterion is met. Three different stopping criteria (or any combination among them) may be used in current saCeSS2 implementation: maximum number of evaluations, maximum execution time and a *value-to-reach* (*VTR*). While the *VTR* is usually known in benchmark problems, for a new problem, the *VTR* will be, in general, unknown.

## Applications in computational systems biology

The aim of reverse engineering in biological systems is to infer, analyze and understand the functional and regulatory mechanisms that govern their behavior, using the interplay between mathematical modeling with experiments. Most of this models need to explain dynamic behavior, so they are usually composed of different types of differential equations. However, reverse engineering in systems biology has to face many pitfalls and challenges, especially regarding the ill-conditioning and multimodality of these inverse problems [10]. Below we consider several cases related with cell signalling processes and show how these issues can be surmounted with the methodology presented here.

### Reverse engineering of cell signalling

Reverse engineering of cell signaling phenomena is a particularly important area in systems biology [64]. In complex organisms, signaling pathways play a critical role in the behavior of individual cells and, ultimately, in the organism as a whole. Cells adapt to the environmental conditions through the integration of signals released by other cells via endocrine or paracrine secretion as well as other environmental stimuli. Fundamental cellular decisions such as replicate, differentiate or die (apoptosis) are largely controlled by these signals [65].

Many of the interactions involved in signaling are commonly grouped in pathways. Pathways are typically depicted as sequences of steps where the information is relayed upon activation by an extracellular receptor promoting several downstream post translational modifications, which will ultimately end by modifying gene expression or some other effector. These interactions are dynamic, i.e. the behavior of such pathways is known to be highly dependent on the cell type and context [66], which change with time [67]. Additionally, many of these pathways interact with each other in ways that are often described as analog to a decision making process [68]. Further, the dynamics of cell signaling are rather fast processes, specially if compared with metabolism or even gene expression.

There are at least three good reasons to infer a dynamic model of a signaling pathway. The first, and perhaps most obvious one, is to find novel interactions. The second is model selection, defined as the process of using data to select (or exclude) a number of model features which are consistent with the current knowledge about a given system. This is particularly relevant when comparing different cell types or a specific cell type in its healthy and diseased status, such as cancer. The third one is the usage of such a model to predict how the system will behave in new conditions that have not been tested before.

In order to build a mechanistic dynamic model for a given cell type or tissue, we need values for its parameters. These are rarely available, and a common strategy is to find them by training the model to data. The most informative data for signal transduction is obtained upon perturbation experiments, where typically a system (assumed to be homeostatic initially) is stimulated with different chemicals to which the cell may (or not react), and the variations in the cell biochemistry are recorded. The resulting time-series of data are then used to reverse engineer a dynamic model of the signalling process.

Following subsections describe the so called logic-based ordinary differential equations (ODE) framework, which has been found particularly useful in modeling cell signalling, and its problem statement as a MIDO. Then, in the results, we present three very challenging case studies of increasing complexity, which are then solved with the parallel metaheuristic presented in this study.

### Logic-based dynamic models

Logic models were first applied to biological systems by [69] to model gene regulatory networks. Since then, applications to multiple contexts have been made [70, 71] and diverse modifications from the original formalism have been developed [72]. In particular, various extensions have been developed to accommodate continuous values (e.g. [32, 73–78]). Amongst these formalisms, logic-based ODEs are one of the best options to handle time series with precision [33, 77]. The main idea is to convert the logic models into their continuous ODE-based equivalent but without the need of mechanistic (kinetic information). However, since it is composed of differential equations, we can use it to carry out dynamic simulations and e.g. predict dynamic trajectories of variables of our interest. A number of different methods have been proposed transform Boolean logic models into ODE homologues [74, 75, 77].

Basically, Boolean models describe the flow of information in a biological system using discrete binary states (logic decisions). In other words, each state *x*_i_ ∈ {0, 1} is represented by a binary variable can be updated according to a Boolean function *B*_i_(*x*_i1_, *x*_i2_, …, *x*_iN_) ∈ {0, 1} of its *N* inputs (*x*_ij_). A typical simple example is the situation where a protein can be phosphorylated in two sites by different kinases, and both interactions are needed to activate the protein. This can be modeled as a logic AND gate. Alternatively, when two different kinases can phosphorylate the same site, independently activating the downstream signaling, we can describe it as a logic OR gate. In another situation, if a signal inhibits the propagation of another one, we can describe it with a NOT gate. In summary, logic models can be represented by an hypergraph with AND/OR/NOT gates.

In the logic-based ODE formalism, we transform each Boolean update function into a continuous equivalent ${\bar{B}}_{i}\in \left[0,1\right]$, where the states ${\bar{x}}_{i}\in \left[0,1\right]$ can take continuous values between 0 and 1. Their dynamic behaviour is then modelled as:

$$\begin{array}{c} {\dot{\bar{x}}}_{\text{i}}=\frac{1}{\tau_{\text{i}}}\cdot \left({\bar{B}}_{\text{i}}\left({\bar{x}}_{\text{i}1},{\bar{x}}_{\text{i}2},\ldots ,{\bar{x}}_{\text{ij}}\right)-{\bar{x}}_{\text{i}}\right) \end{array}$$ (7)

where *τ*_i_ can be regarded as a sort of life-time of *x*_i_.

HillCubes [77] have been developed, based on multivariate polynomial interpolation, for the above purpose. They include Hill kinetics (which are known to provide a good approximation of the dynamics of e.g. gene regulation). HillCubes are obtained via a transformation method from the Boolean update function. An example is shown in Table 1, illustrating how an OR gate would be transformed by multi-linear interpolation [77] into a BoolCube (${\bar{B}}^{I}$):

$$\begin{array}{c} \begin{array}{cc} {\bar{B}}^{I} & ({\bar{x}}_{1},\ldots ,{\bar{x}}_{N})=\\ & \sum_{x_{1}=0}^{1}\ldots \sum_{x_{N}=0}^{1}[B(x_{1},\ldots ,x_{N})\cdot \prod_{i=1}^{N}(x_{i}{\bar{x}}_{i}+\left[1-x_{i}\right]\left[1-{\bar{x}}_{i}\right])] \end{array} \end{array}$$ (8)

**Table 1:** Relation between functions *B*(*x*_1_, *x*_2_) and ${\bar{B}}^{I}\left({\bar{x}}_{1},{\bar{x}}_{2}\right)$.

*x*_1_	*x*_2_	*B*(*x*_1_, *x*_2_)	${\bar{B}}^{I}\left({\bar{x}}_{1},{\bar{x}}_{2}\right)=\ldots$
0	0	0	$0\cdot \left(1-{\bar{x}}_{1}\right)\cdot \left(1-{\bar{x}}_{2}\right)+$
0	1	1	$1\cdot \left(1-{\bar{x}}_{1}\right)\cdot {\bar{x}}_{2}+$
1	0	1	$1\cdot {\bar{x}}_{1}\cdot \left(1-{\bar{x}}_{2}\right)+$
1	1	1	$1\cdot {\bar{x}}_{1}\cdot {\bar{x}}_{2}$

A truth table helps to understand the relationship between the OR Boolean update function *B*(*x*_1_, *x*_2_) and its continuous homologue ${\bar{B}}^{I}\left({\bar{x}}_{1},{\bar{x}}_{2}\right)$. For every combination of the Boolean variables *x*_1_ and *x*_2_, a term is added to ${\bar{B}}^{I}\left({\bar{x}}_{1},{\bar{x}}_{2}\right)$) depending on *B*(*x*_1_, *x*_2_).

Although BooleCubes are accurate homologues of Boolean functions, they fail to represent the typical sigmoid shape switch-like behavior often present in molecular interactions [79]. The latter can be achieved by replacing the ${\bar{x}}_{i}$ by a Hill function:

$$\begin{array}{c} f^{H}\left({\bar{x}}_{i}\right)=\frac{{{\bar{x}}_{i}}^{n}}{{{\bar{x}}_{i}}^{n}+k^{n}} \end{array}$$ (9)

or the normalized Hill function:

$$\begin{array}{c} f^{Hn}\left({\bar{x}}_{i}\right)=\frac{f^{H}\left({\bar{x}}_{i}\right)}{f^{H}\left(1\right)} \end{array}$$ (10)

Further details regarding logic-based ODE models can be found in [77].

### Problem statement as a MIDO

In order to find the best logic-based dynamic model to represent the behavior of a given biological network, we developed a formulation extending previous works that used a Boolean logic framework [80] or a constrained fuzzy-logic formalism [81]. The idea here is that starting from a directed graph containing only the interactions and their signs (activation or inhibition) we can build an expanded hypergraph containing all the possible logic gates.

The problem can be formulated as the following for case studies 1 and 2:

$$\begin{array}{c} \begin{array}{cccc} & \underset{n,k,\tau ,w}{\text{minimize}} & & F\left(n,k,\tau ,w\right)=\sum_{\epsilon =1}^{n_{\epsilon }}\sum_{o=1}^{{n_{\text{O}}}^{\epsilon }}\sum_{s=1}^{{n_{\text{S}}}^{\epsilon ,o}}{\left({{\tilde{y}}_{\text{S}}}^{\epsilon ,o}-{y_{\text{S}}}^{\epsilon ,o}\right)}^{2}\\ & \text{subject}\;\text{to} & & E_{\text{sub}}=\left\{e_{\text{i}}|w_{\text{i}}=1\right\},\;i=1,\ldots ,n_{\text{hyperedges}}\\ & & & H_{\text{sub}}=(V,E_{\text{sub}})\\ & & & {\text{LB}}_{n}\leq n\leq {\text{UB}}_{n}\\ & & & {\text{LB}}_{k}\leq k\leq {\text{UB}}_{k}\\ & & & {\text{LB}}_{\tau }\leq \tau \leq {\text{UB}}_{\tau }\\ & & & \dot{\bar{x}}=f\left(H_{\text{sub}},\bar{x},n,k,\tau ,t\right)\\ & & & \bar{x}\left(t_{0}\right)={\bar{x}}_{0}\\ & & & y=g(H_{\text{sub}},\bar{x},n,k,\tau ,t) \end{array} \end{array}$$ (11)

where $H_{\text{sub}}$ is the subgraph containing only the hyperedges ($E_{\text{sub}}$), defined by the binary variables *w*. Additionally *n*, *k* and *τ* are the continuous variables required for the logic-based ODE scheme. Upper and lower bounds represent the limits to these parameters. The model dynamics ($\dot{\bar{x}}$) are given by the function *f*. This set of differential equations varies according to the subgraph (and therefore also according to the integer variables vector *w*). Predictions for the systems dynamics are obtained by solving the initial value problem given by the ODEs. The objective function is the mismatch (e.g. norm-2) between the simulated (*y*) and the experimental data ($\tilde{y}$), and we seek to minimize this metric for every experiment (*ϵ*), observed species (*o*) and sampling point (*s*). The simulation data *y* is given by an observation function *g* of the model dynamics at time *t*.

In case study 3 we also consider a model reduction problem where additional decision variables are used to remove the influence of a regulator ${\bar{x}}_{i}$ from the model. As starting point we consider a model derived with SELDOM [82], where a mutual information strategy, combined with dynamic optimization, was used to find an ensemble of dynamic models that can explain the data from four breast-cancer cell-lines used in the DREAM-HPN challenge [83]. One of the critical steps in SELDOM was to perform model reduction using a greedy heuristic. Here we consider instead the application of mixed-integer global optimization with saCeSS2 to the problem of model reduction. To find a reduced model we use the Akaike information criterion (AIC), which for the purpose of model comparison is defined as:

$$\begin{array}{c} AIC=2K+2n\cdot ln(\frac{F}{n}), \end{array}$$ (12)

where *K* is the number of active parameters. The theoretical foundations for the AIC can be found in [84].

## Results and discussion

The new saCeSS2 method described above has been applied to a set of case studies from the domain of computational systems biology with the goal of assessing its efficacy and efficiency for solving these very difficult MIDO/MINLP problems. The method has been compared with both the sequential eSS [31] and with an embarrassingly parallel non-cooperative version of the eSS called *np*-eSS. The *np*-eSS method consists of *np* separated eSS runs performed in parallel but without cooperation among them. The results reported for *np*-eSS correspond to the best value achieved in *np* runs. Diversity is introduced in these *np* eSS runs by allowing different settings to each one of the individual searches. The performance of saCeSS2 was also evaluated considering a different number of processors in order to study its scalability and the dispersion of results.

The original reported implementation of eSS [31] was coded in Matlab, thus, for a fair comparison with saCeSS2, it has been here implemented in F90. In the saCeSS2 algorithm the MPI library [85] has been employed for the cooperation between islands.

For the experimental testbed different platforms have been used. First, most of the experiments were conducted in a local cluster (NEMO) that consists of three nodes powered with two deca-core Intel Xeon E5-2650 CPUs with 30GB of RAM connected through a Gigabit Ethernet network. With the aim of assessing the scalability of the proposal we also performed some experiments in a larger infrastructure, the cluster from European Bioinformatics Institute (EBI) [86], that consists of 222 nodes powered with two octa-core Intel Xeon E5-2680 CPUs with 30GB of RAM, connected through a Gigabit Ethernet network. Finally, in order to evaluate the performance of the proposal in a public cloud, some experiments were conducted in the Microsoft Azure public cloud.

The saCeSS2 library has been compiled with the Intel implementations for C, FORTRAN and MPI library, except in the EBI Cluster, where GNU compilers and openMPI had to be used. We remark this fact due to the well-known differences in the performance obtained using different compilers.

The computational results shown in this paper were analyzed both from a horizontal view [87], that is, assessing the performance by measuring the time needed to reach a given target value, and from a vertical view [87], that is, evaluating how far a method has advanced in the search for a predefined effort. Thus, two different stopping criteria were considered in these experiments: solution quality based on a *value-to-reach* (*VTR*), for an horizontal view, and predefined effort using a maximum execution time, for a vertical approach. The *VTR* used was the optimal fitness value reported in [33]. Also, due to the natural dispersion in the results of stochastic methods, each experiment reported in this section has been performed 20 times and a statistical study was carried out.

### Case study 1: Synthetic signaling pathway (SSP)

The synthetic signaling pathway (SSP) [72] case study considers a dynamic model composed of 26 ordinary differential equations and 86 continuous parameters. It was initially used to illustrate the capabilities and limitations of different formalisms related with logic-based models. Although this is a synthetic problem, it was designed to be a plausible representation of a signaling transduction pathway. The model was used to generate pseudo-experimental data for 10 combinations of perturbations with two extracellular ligands (TNF and EGF) and two kinase inhibitors (for PI3K and RAF1). From a total of 26 dynamic states, 6 were observed (NFKB, P38, AP1, GSK3, RAF1 and ERK) and 5% of Gaussian noise was added to the data.

Following the methodology described in [80], we obtained an expanded version of this model containing every possible AND/OR logic gate given the initial graph structure. This so-called expansion procedure generated a nested model comprising 34 additional variables, one for each hyperedge. Thus, the obtained optimization problem contains 120 parameters, being 86 continuous and 34 binaries. We proceeded by implementing the model and experimental setup using AMIGO [88] and exporting C code which could be used with the saCeSS2 method presented here.

Considering saCeSS2, it is important to note that the cooperation between processes changes the systemic properties of the eSS algorithm and therefore its macroscopic behavior. The same happens with the self-adaptive mechanism proposed. Table 2 displays for each method (sequential, parallel non-cooperative, and saCeSS2) the number of processors used (#np), the mean and standard deviation value of the achieved tolerances (*fbest*), the mean and standard deviation number of external iterations (*iter*) performed, the mean and standard deviation number of evaluations (*evals*) needed to achieve the *VTR*, the mean and standard deviation execution time, and the speedup achieved versus the sequential method. As it can be seen, there is a notable reduction in the execution time required by the parallel methods against the sequential one, and there is also a significant reduction between the saCeSS2 method and the non-cooperative *np*-eSS. Note that, in the parallel methods (*np*-eSS and saCeSS2), the initial population, and, thus, the computational load, is not spread among processors. The population size is the same in the sequential method as in each of the islands in the parallel methods. That is, the parallel methods allow for a diversification in the search. Therefore, the speedup achieved versus the sequential method is due to the impact of this diversification, and the speedup achieved by saCeSS2 over the *np*-eSS is due to the impact of the cooperation between different searches, that produces results of higher quality performing less evaluations and, hence, providing a better performance. In short, these results show the effectiveness of the cooperative parallel algorithm proposed compared to a non-cooperative parallel version.

**Table 2:** Case study 1 SSP: Performance analysis from a horizontal view.

method	#np	mean fbest±std	mean iter±std	mean evals±std	mean time±std(s)	speedup
eSS	1	9.8±0.3	261±636	345989±829560	54885±131153	-
*np*-eSS	10	9.5±0.6	35±16	356583±127682	4546±1592	12.0
	20	9.8±0.2	29±6	626150±120338	4193±907	13.0
	40	9.8±0.1	33±9	876800±228596	2901±765	18.9
saCeSS2	10	9.7±0.2	25±9	246082±68925	3478±1114	15.7
	20	9.8±0.1	18±6	402613±120260	2779±870	19.7
	40	9.9±0.1	19±8	470746±142702	1602±523	34.2

Performance of the saCeSS2 and scalability analysis when the number of processors grows. Stopping criteria: VTR = 10.

Table 3 shows results for experiments that include as stopping criterion a predefined effort of maximum execution time of 4000 seconds. This table displays the percentage of executions (% hit) that achieve a very high quality solution (VTR = 9.0). It can be observed that the sequential implementation never achieved the VTR in the maximum allowed time, while, for the parallel implementations, when the number of processes grows the number of the executions that achieved the quality solution increased. Again, the cooperative proposed saCeSS2 implementation achieved better results than the non-cooperative parallel version when using the same number of processors.

**Table 3:** Case study 1 SSP: Performance analysis from a vertical perspective.

method	#np	mean fbest±std	mean iter±std	mean evals±std	mean time±std(s)	hits%
eSS	1	20.1±4.5	19±3	27289±4478	4000±0	0%
*np*-eSS	10	10.6±1.6	26±3	261664±24780	3966±119	10%
	20	10.31±0.91	24±3	522194±47322	3862±410	15%
	40	9.5±0.7	36±4	964168±99650	3681±510	30%
saCeSS2	10	10.2±1.4	23±4	256872±28063	3822±476	15%
	20	9.6±0.7	22±4	471948±82282	3532±706	35%
	40	8.9±0.2	32±19	621118±232204	2258±973	85%

Performance of saCeSS2 using as stopping criteria: VTR = 9 and maximum time = 4000 seconds.

**Fig 3:**
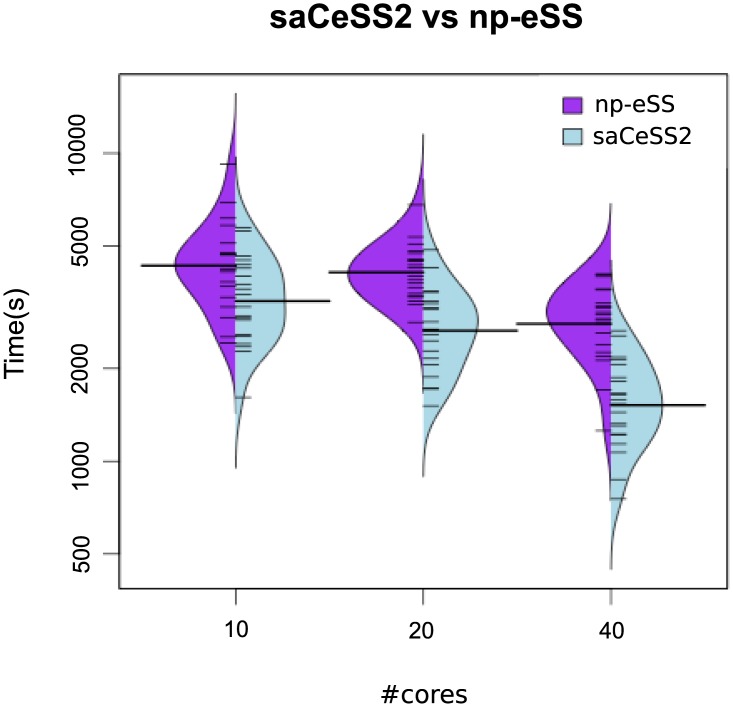
Case study 1 SSP: Bean plots of execution time for *np*-eSS vs saCeSS2 using 10, 20 and 40 MPI processors. VTR = 10 and 20 independent runs for each experiment.

When dealing with stochastic optimization solvers, it is important to evaluate the dispersion of the computational results. Fig 3 illustrates with beanplots how the parallel algorithms (*np*-eSS and saCeSS2) reduce the variability of execution time and obtain less number of outliers when the number of cores increases. Hybrid violin/boxplot graphs for these results are included in Fig A in S1 File for a in depth insight. The proposed saCeSS2 method outperforms significantly the non-cooperative *np*-eSS method (note the logarithmic scale in axis y). This is an important feature of the saCeSS2, because it reduces the average execution time.

**Fig 4:**
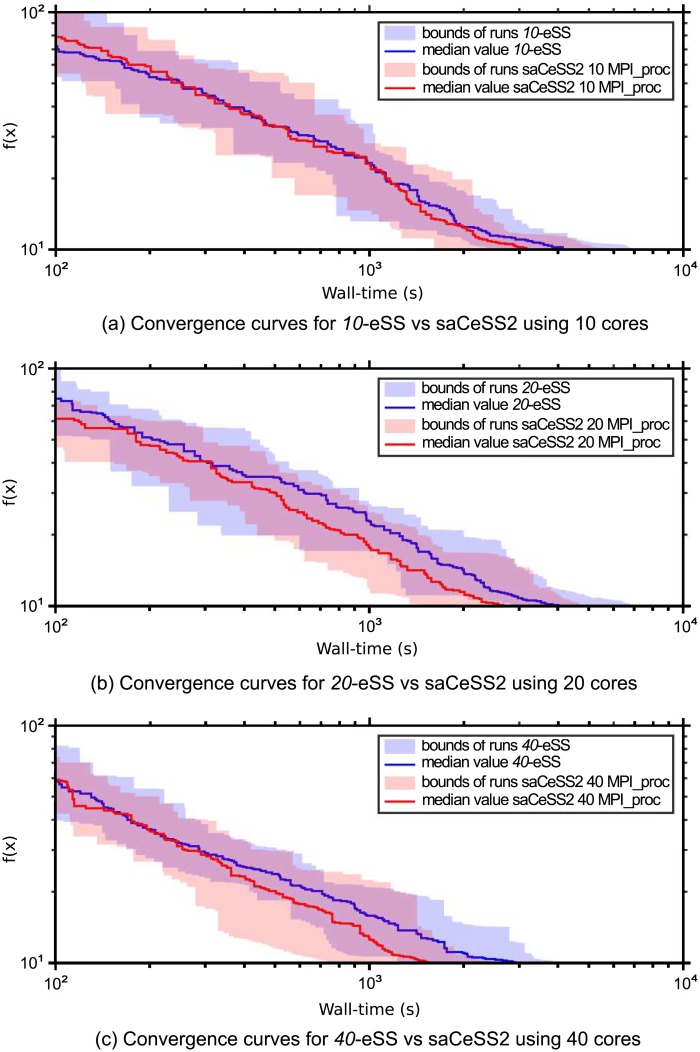
Case study 1 SSP: Convergence curves.

To better illustrate the goal of saCeSS2 method vs the non-cooperative parallel *np*-eSS implementation, Fig 4 shows the convergence curves, which represent the logarithm of the objective function value against the execution time. Fig 4 illustrates, for both saCeSS2 and *np*-eSS methods, the region between the lower and upper bounds of the 20 runs performed for each experiment, with a strong line representing the median value for each time moment.

**Fig 5:**
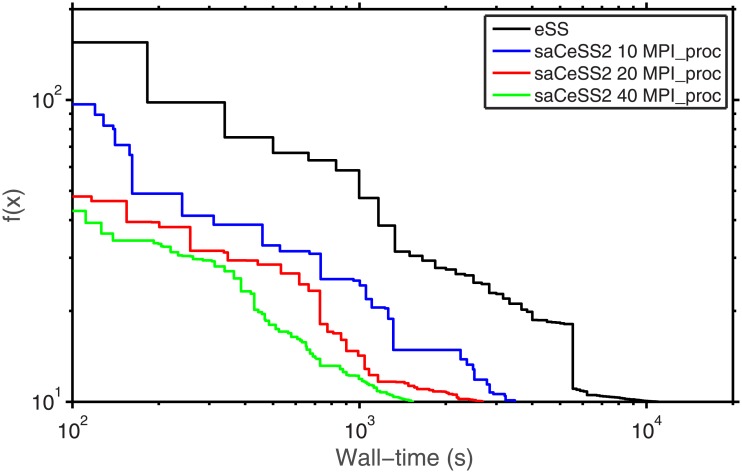
Convergence curves for saCeSS2 using 1, 10, 20 and 40 processors corresponding to the runs in the median values of the results distribution.

In order to evaluate the scalability of the proposed saCeSS2, Fig 5 shows the convergence curves for those experiments that fall in the median values of the results distribution using 10, 20 and 40 processors. It can be seen that the saCeSS2 still improves the convergence results when the number of processors grows. This improvement comes from the cooperation between islands and the diversification obtained through the exploration in parallel of different search regions using different algorithm settings.

### Case study 2: HepG2

As a second case study, we consider the reverse engineering of a logic-based ODE model using liver cancer data (a subset of the data generated by [89]). The dataset consists of phosphorylation measurements from a hepatocellular carcinoma cell line (HepG2) at 0, 30 and 180 minutes after perturbation.

To preprocess the network, we used CellNOptR, the R version of CellNOpt [90]. Basically, the network was compressed to remove as many non-observable/non-controllable species as possible. Subsequently, we generated all gates that were compatible with the network; for this we added hyperedges (AND gates) from all pair of inputs (the OR gates are implicit). The expanded network has 109 hyperedges and 135 continuous parameters. To transform this model into a logic-based ODE model, we developed a parser that generates a C model file and Matlab scripts compatible with the AMIGO toolbox [88].

Consequently, in this case the optimization problem to solve contains a total of 244 parameters, being 135 continuous and 109 binaries. Although the time-series data contains only three sampling time points, it is quite rich from the point of view of information content: it includes 64 perturbations comprising 7 ligands and 7 small-molecule inhibitors. The ligands were chosen to activate inflammation and proliferation pathways, and the inhibitors to block the activity of specific kinases. We normalized the data by rescaling it to this range. This is required to as the models are semi-quantitative and hence the data has to be between 0 and 1. There are a total of 25 states present in the model, 16 corresponded to observed species. The initial conditions for the other 9 species are not measured and we had to estimate them. To avoid increasing the problem size and multi-modality unnecessarily, the estimated initials conditions were assumed the same for each of the 64-experiments.

Table 4, similarly to Table 2, displays the performance of the different methods based on the number of external iterations, function evaluations and total execution time, for a different number of processors. Note that results for the sequential method are not reported due to the unreasonable amount of time to reach convergence. Again, it can be seen that the saCeSS2 method outperforms, not only the sequential eSS, but also a parallel eSS without cooperation between islands. The cooperative strategy, along with the self-adaptive mechanism, leads to an important improvement in the convergence rate and the execution time required.

**Table 4:** Case study 2 HepG2: Performance analysis from a horizontal view.

method	#np	mean fbest±std	mean iter±std	mean evals±std	mean time±std(s)
eSS	1	-	-	-	-
*np*-eSS	10	32.4±0.8	1493±2975	13581782±10598705	230483±365129
	20	32.5±0.7	527±381	20267424±12791177	142996±93617
	40	32.8±0.1	434±246	22157687±11660663	70221±35565
saCeSS2	10	32.5±0.5	1056±1873	13637565±20933642	167880±242658
	20	32.3±0.9	396±496	13196677±15577101	89433±108108
	40	32.4±1.0	560±431	11959105±9383238	44037±32346

Performance of the saCeSS2 and scalability analysis when the number of processors grows. Stopping criteria: VTR = 33.

Table 5 shows results including as stopping criterion a lower VTR and a predefined effort of 30 hours. Since it is very difficult to reach a point of very high quality in this problem, this table displays the percentage of hits that achieve a VTR = 30. It can be observed that the sequential eSS never achieved the VTR in the maximum allowed time, while the parallel implementations achieve more hits as the number of processors grows. The saCeSS2 method clearly outperforms the embarrassingly parallel eSS: not only the mean time improves (around a 67% for 40 processors), but, which is more important, the number of runs that achieve the high quality VTR is larger (65% versus 35% for 40 processors).

**Table 5:** Case study 2 HePG2: Performance analysis from a vertical view.

method	#np	mean fbest±std	mean iter±std	mean evals±std	mean time±std(s)	hits%
eSS	1	48.3±6.2	342±47	1010744±122417	108000±0	0%
*np*-eSS	10	34.9±3.7	482±112	8329751±1519410	103847±14515	10%
	20	32.9±1.9	418±65	16612721±2292759	103614±14763	10%
	40	31.1±1.3	604±180	30606532±8576193	93874±25243	35%
saCeSS2	10	34.7±3.8	403±146	7359986±1468640	103052±12461	10%
	20	32.0±4.4	339±163	12057460±4431722	85436±28200	45%
	40	30.4±1.2	786±478	20231060±11664025	63153±34832	65%

Convergence of saCeSS2 until a high quality solution is reached. Stopping criteria: VTR = 30 and maximum time = 108000 seconds.

**Fig 6:**
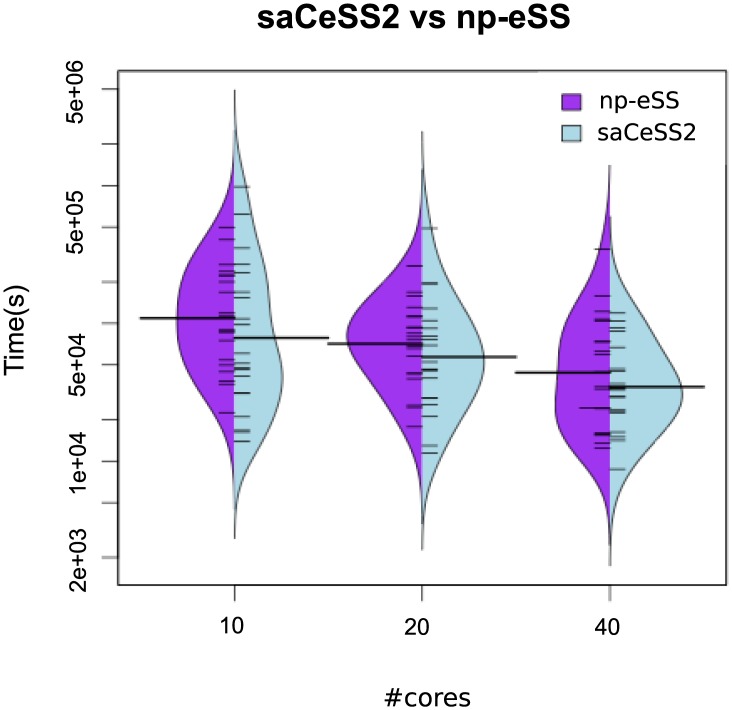
Case study 2 HePG2: Bean plots of execution time for *NP*-eSS vs saCeSS2 using 10, 20 and 40 MPI processors in the HePG2 problem. VTR = 33 and the number of runs is equal to 20.

Fig 6 shows beanplots comparing the distribution of the execution times in the saCeSS2 method versus the non-cooperative parallel version. The figure illustrates not only the improvement in the mean execution time, but also the reduction in the variability of the execution times due to the cooperation and self-adaptive mechanism included in the saCeSS2 method. Hybrid violin/boxplots for this data are also provided in Fig B in S1 File for a thoroughly comprehension. Notice that the less the number of cores used the more outliers we obtain in the distribution.

**Fig 7:**
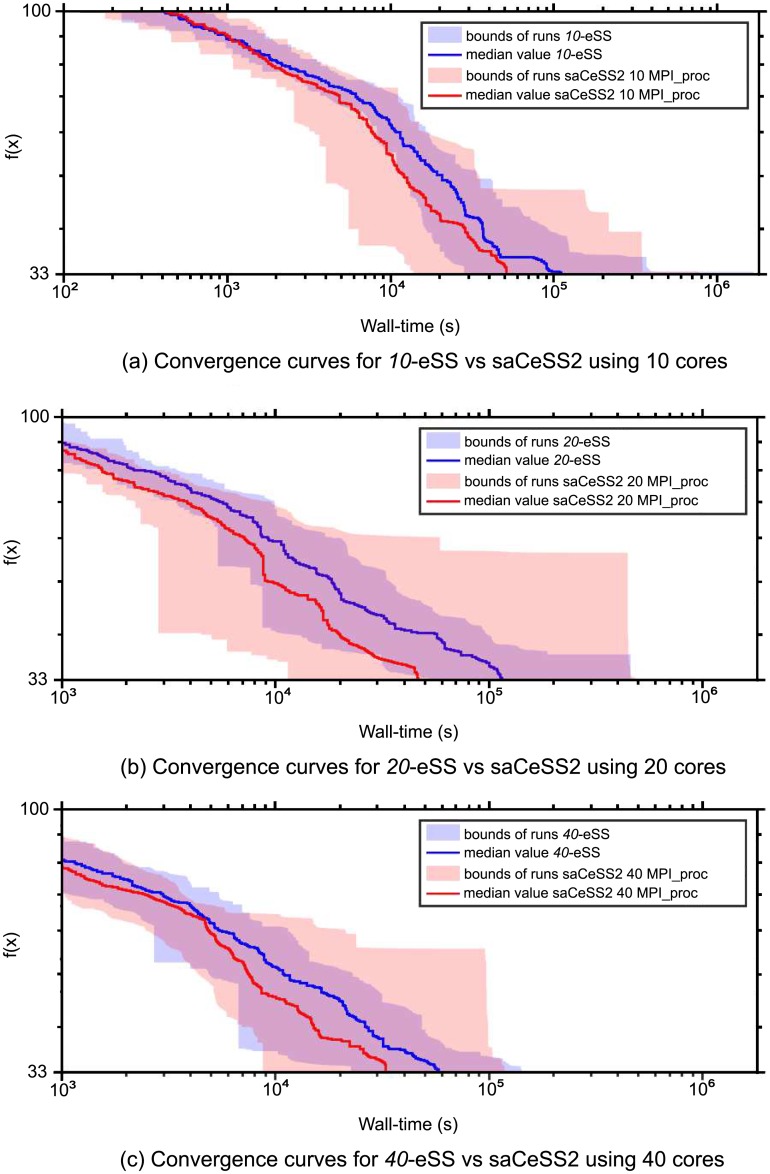
Case study 2 HePG2: Convergence curves.

**Fig 8:**
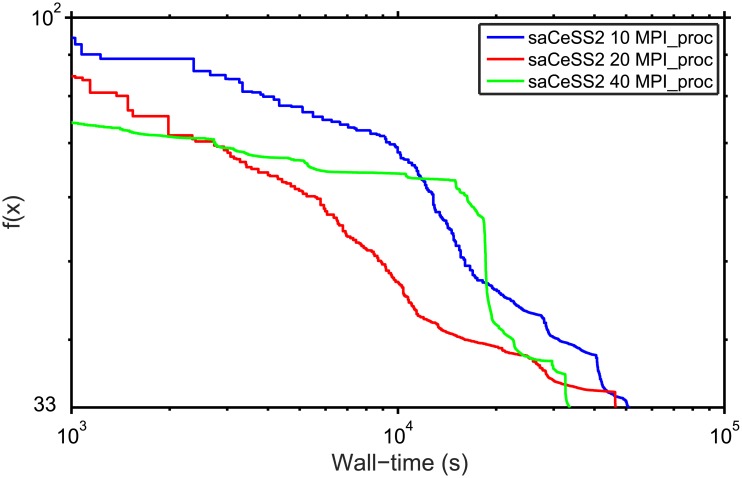
Convergence curves for saCeSS2 using 10, 20 and 40 processors corresponding to the runs in the median values of the results distribution.

Finally, Fig 7 shows the convergence curves for the previous experiments. Fig 7 shows the region between the lower and upper bounds of the 20 runs for each experiment. Fig 8, demonstrate the scalability of the proposal when the number of processors grows.

### Case study 3: Breast cancer network inference challenge (HPN-DREAM)

We finally consider an extremely difficult problem which has been recently made publicly available in the context of the DREAM challenges [91]. The DREAM challenges provide a forum to crowdsource fundamental problems in systems biology and medicine, such as the inference of signaling networks [83, 92], in the form of collaborative competitions. This data-set comprised time-series acquired under eight extracellular stimuli, under four different kinase inhibitors and a control, in four breast cancer cell lines [83].

The HPN-DREAM breast cancer challenge is composed of two sub-challenges: (i) an experimental sub-challenge where the participants were asked to make predictions for 44 observed phosphoproteins (although the complete data-set was larger); and (ii) an *in silico* sub-challenge, where the participants were encouraged to exploit all the prior knowledge they could use and the experimental protocol along with the real names of the measured quantities, used reagents, inhibitors, etc. Using different combinations of inhibitors and ligands (on and off), the organizers if the challenge generated a data-set for several cell-lines. An additional data-set generated with the help of a fourth inhibitor was kept unknown to the participants, who were asked to deliver predictions for several possible inhibitors.

**Fig 9:**
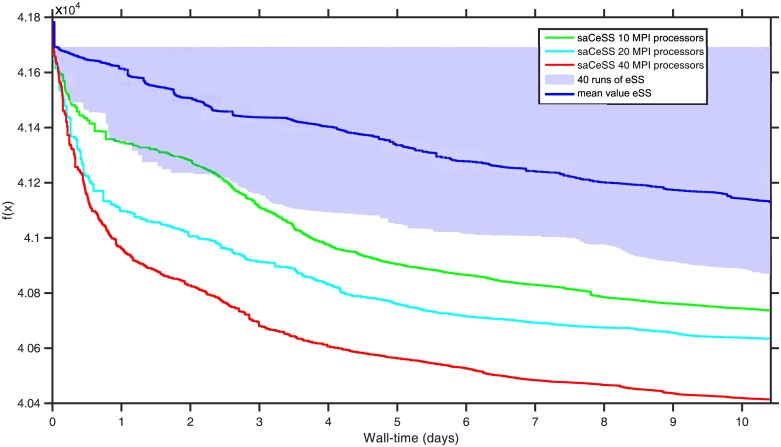
Case study 3: HPN-DREAM convergence curves in the NEMO local cluster. Convergence curves using 10, 20, 40 and 60 cores.

**Fig 10:**
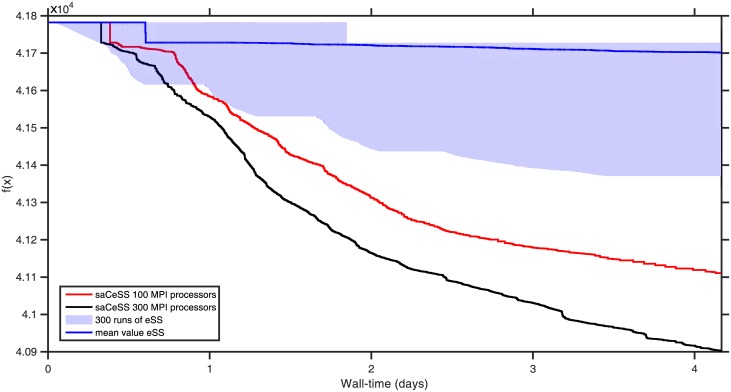
Case study 3: HPN-DREAM convergence curves in the EBI cluster. Convergence curves using 100, and 300 cores.

Overall, the problem contains a total of 828 decision variables (690 continuous and 138 binaries). Thus, the HPN-DREAM is an extremely challenging problem also from the computational view, with an enormous expected execution time and an unknown final target value. In a preliminary step, we carried out different experiments using *np* = 10, 20, and 40 cores in our NEMO local cluster to solve this problem. We used the *np* cores to run in parallel *np* independent eSS searches, without cooperation between them, and we also run a saCeSS2 execution using *np* processes. We used as stopping criterion for all the experiments a predefined effort of 10 days and we studied the convergence curves (shown in Fig 9). The blue region represents the bounds of the 40 sequential eSS runs, while the blue solid line represents the median value for each time moment of these 40 runs. The other solid lines represent the convergence curve of a single saCeSS2 performed using 10, 20, and 40 cores. The saCeSS2 method clearly outperforms the embarrassingly parallel eSS and shows a good scalability when the number of processes increases. We then performed new experiments using a larger number of cores in the EBI cluster. Fig 10 show the convergence curves using 100 and 300 cores. Due to the large amount of resources employed and the cluster policy, the length of the job (and, thus, the stopping criterion used) had to be set to 4 days. Note that, due to the differences between both infrastructures, it is quite difficult to perform a fair comparison with our local cluster. Although the convergence rate seems to be slower in the EBI cluster, the results obtained still demonstrate the good scalability of saCeSS2. The lower convergence rate in the EBI cluster is due to the architectural and performance differences with respect to our local cluster, and also to the use of GNU compilers instead of the Intel compilers used in our local cluster. Nevertheless, the scalability of saCeSS2 is maintained: the more resources we can use for the cooperative method, the larger improvement we will obtain versus executing the sequential method with the same computational resources.

### Performance evaluation of saCeSS2 in the Cloud

As it was already demonstrated in previous subsections, though saCeSS2 clearly outperforms the sequential and the non-cooperative parallel versions of the eSS, it still requires large computational times to achieve convergence in very hard problems. Additionally, it has been shown that the diversity introduced by the increase in the number of islands clearly improves the algorithm convergence rate. However, an increase in the number of islands should be attended by an increase in the number of computational resources (cores), and this is not always practicable.

With the advent of Cloud Computing, effortless access to a large number of distributed resources has become more feasible. However, the scientific computing community has been quite hesitant in using the Cloud, because traditional programming models do not fit well with the new paradigm. In the last decade, several researchers have studied the performance of HPC applications in the cloud environment [93–97]. Most of these studies use classical MPI benchmarks to compare the performance of MPI on public cloud platforms. These works conclude that the lack of high-bandwidth, low-latency networks, as well as the virtualization overhead, has a large effect on the performance of MPI applications on the cloud. In this section we explore the use of a cloud platform, the Microsoft Azure public cloud, for deploying saCeSS2 experiments. The performance was compared to the one obtained in the NEMO local cluster in terms of computational time. Finally, the cost of cloud resources were also analyzed. Thus, this study could be useful for those researchers interested in the performance of traditional parallel metaheuristics in new cloud platforms and its market price.

Some of the previous experiments were deployed in the Microsoft Azure public cloud using clusters with compute-intensive A9 instances (16 cores, 112GB). These instances are designed and optimized for compute-intensive and network-intensive applications. Each A9 instance uses an Intel Xeon E5-2670 @2.6GHz CPUs with 112GB of RAM. Additionally, A9 instances feature a second network interface for remote direct memory access (RDMA) connectivity. This interface allows instances to communicate with each other over an InfiniBand network, operating at QDR rates, boosting the scalability and performance of many MPI applications.

Table 6 shows the performance of the saCeSS2 method for both SSP and HePG2 case studies in the Azure public cloud. As it can be seen the behavior of the algorithm differs slightly from the results obtained in the NEMO local cluster and reported in previous subsections. In particular, results for a small number of processors are better in Azure than in the local cluster. However, the results obtained in the local cluster outperforms the ones in Azure when the number of processors grows. In particular, note that the number of function evaluations required for convergence is larger in the experiments carried out in the local cluster than in the same experiments carried out in Azure when the number of processors is small (10 cores), and it is the opposite for the experiments that use 20 and 40 cores. This can be attributed to the efficiency of the inter-node communications (remember that each Azure instance has 16 cores). The higher latency in the inter-node communications in Azure leads to a slow propagation of promising results between islands, that results in a slower convergence.

**Table 6:** Performance of saCeSS2 for both SSP and HePG2 case studies in azure public cloud.

problem	#np	mean fbest±std	mean iter±std	mean evals±std	mean time±std(s)	mean price
SSP	10	9.8±0.2	23±8	246256±73545	3153±948	1.99 €
	20	9.8±0.2	21±9	470857±175723	3057±1177	1.93 €
	40	9.8±0.1	31±44	571966±423381	1861±1426	1.18 €
HePG2	10	32.6±0.3	807±782	11790096±10574631	151939±128256	96.10 €
	20	32.0±1.2	305±243	10617112±7403497	87802±58619	55.53 €
	40	32.8±0.3	731±707	17438937±19853336	68214±77442	43.14 €

Stopping criteria: *VTR*_*SSP*_ = 10 and *VTR*_*HePG*2_ = 33.

**Fig 11:**
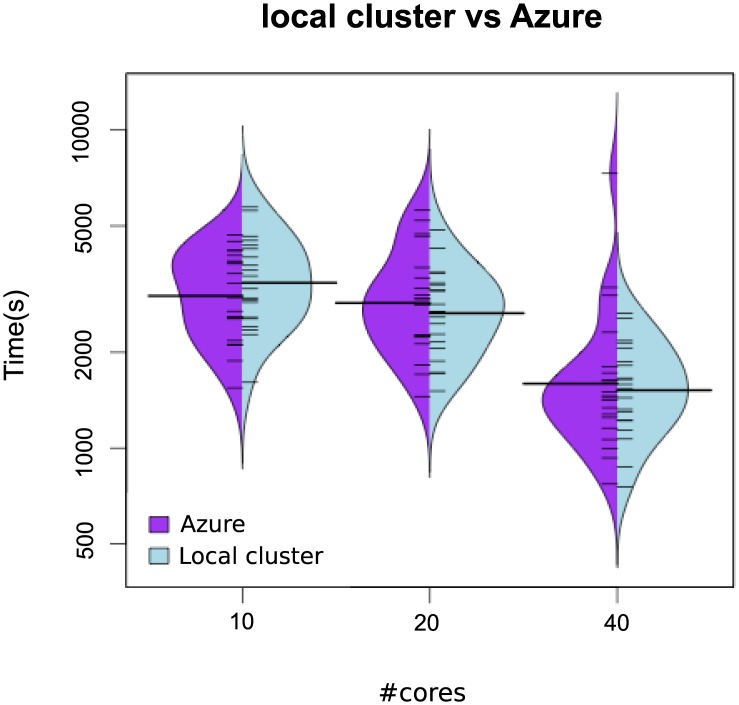
Case study 1: Beanplots comparing results in terms of execution time in azure vs local cluster in the SSP problem.

**Fig 12:**
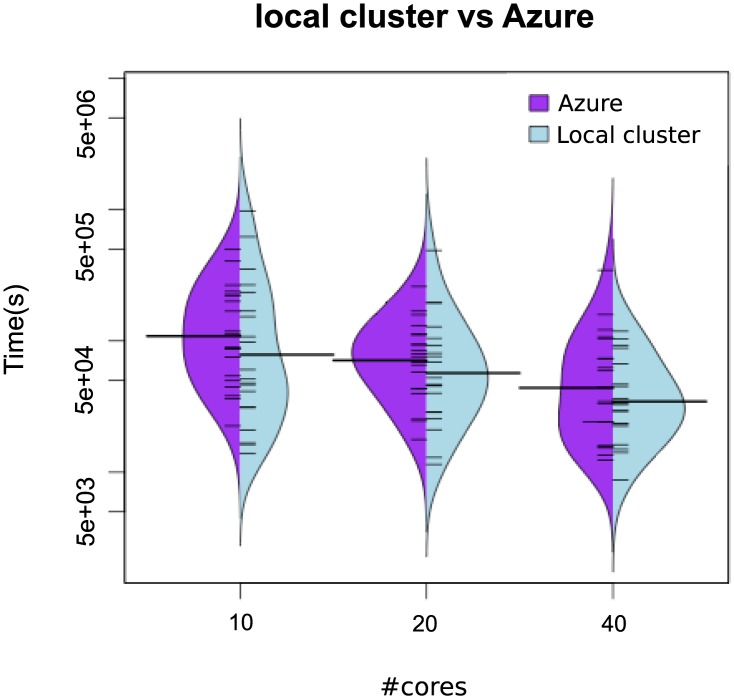
Case study 2: Beanplots comparing results in terms of execution time in azure vs local cluster in the HePG2 problem.

Besides, it is noteworthy that the dispersion in the distribution of the results is larger for experiments carried out in the Azure public cloud, specially when the number of cores grows. Figs 11 and 12 illustrate with beanplots this fact. Note the logarithmic scale in axis y. As it can be seen, the number of outliers increases with the number of cores in Azure. Notice that this is exactly the opposite behavior than in the local cluster, and it can be explained by the virtualization overhead in Azure and the use of non-dedicated resources in a multi-tenant platform. Hybrid violin/boxplots, provided in Fig C in S1 File, contribute to illustrate this issue.

To conclude this evaluation we have found it interesting to carry out a brief study on the cost of these experiments in the Azure public cloud. Conducting a cost analysis comparing the cost of relying on cloud computing and that of owning an in-house cluster would be of particular interest, although is a very difficult task [98]. The acquisition and operational expenses have to be used in estimating the local clusters’ cost. However, the actual cost of local clusters is related to its utilization level. For a local cluster acquired as one unit and maintained for several years, the higher the actual utilization level, the lower the effective cost rate. Besides, labor cost in management and maintenance should also be included, which could be significant. Thus, we found unfeasible an accurate estimation of the cost per hour in our local cluster. Besides, if we take a look to the price of the used instances, we can see that in February 2017 the cost of each A9-instance is 2.2769 EUR/hour. The mean pricing for each experiment is shown in Table 6. In the view of the obtained results we can conclude that, though our experiments in the cloud demonstrates a slightly poorer performance, in terms of execution time, the cloud *pay-as-you-go* model can be potentially a cost-effective and timely solution for the needs of many users.

## Conclusions

In this paper, we present a parallel cooperative strategy for the solution of large mixed-integer dynamic optimization problems. This method, saCeSS2, is based on an parallel enhanced scatter search metaheuristic, with new mechanisms and extensions to handle mixed-integer problems. Our strategy shown good performance results when applied to a set of challenging case studies from the domain of computational systems biology. Further, we performed computational runs in different infrastructures (including a local cluster, a large supercomputer and a public cloud platforms) in order to evaluate latency and scalability issues.

This contribution extends the recently developed saCeSS method [41], a parallel cooperative strategy for non-linear programming (NLP) problems, so that it can successfully solve realistic mixed-integer dynamic optimization (MIDO) problems. To this end, the following features have been included in the new saCeSS2 implementation: (1) an efficient mixed-integer local solver (MISQP), (2) a novel self-adaption mechanism to avoid convergence stagnation, and (3) the injection of extra diversity during the adaptation steps, restarting most of reference set of the reconfigured processes. In the near future, we plan to generalize saCeSS2 one more level, incorporating additional local MINLP solvers [22], and adopting a hyper-heuristic [99] framework to choose and coordinate them.

The computational results for case studies show that the proposal significantly reduces the execution time needed to obtain a reasonable quality solution. Moreover, the dispersion in the obtained results is narrowed when the number of processors grows. These results confirm that the method can be used to reverse engineer dynamic models of complex biological pathways, and indicates its suitability for other applications based on large-scale mixed-integer optimization, such as metabolic engineering [28], optimal drug scheduling [100, 101] and synthetic biology [102].

Finally, although the approach presented here has been developed taking into account the particular class of logic-based ODE models, it can be applied to any model structure that can be parametrized, i.e. that can be defined by a finite set of structural and dynamic parameters. This direction will be explored in future work.

The code and data files needed to reproduce the results reported here at available at:

https://doi.org/10.5281/zenodo.290219.

## Supporting information

S1 File Supplementary info document. (PDF)
