# Supplementary material for: A parallel metaheuristic for large mixed-integer dynamic optimization problems, with applications in computational biology
Source: PLoS One. 2017 Aug 15;12(8):e0182186. doi: 10.1371/journal.pone.0182186 (PMC5557587; doi:10.1371/journal.pone.0182186)
Supplement: S1 File — (PDF) [file pone.0182186.s001.pdf]

A parallel metaheuristic for large mixed-integer nonlinear dynamic optimization problems, with applications in computational biology (supplementary information)

David R. Penas*[1], David Henriques†[1], Patricia González‡[2], Ramón Doallo§[2], Julio Saez-Rodriguez¶[3] and Julio R. Banga‖[1]

[1]BioProcess Engineering Group, IIM-CSIC, Vigo (Spain)
[2]Computer Architecture Group, Universidade da Coruña (Spain)
[3]RWTH Aachen University, Faculty of Medicine, Joint Research Centre for Computational Biomedicine (JRC-COMBINE)

*davidrodpenas@iim.csic.es
†davidh@iim.csic.es
‡patricia.gonzalez@udc.es
§doallo@udc.es
¶saezrodriguez@combine.rwth-aachen.de
‖julio@iim.csic.es

Contents

1 Introduction **2**

A Case study 1 SSP: violin/box plots of execution time for *np*-eSS vs saCeSS2 using 10, 20 and 40 MPI processors. **3**

B Case study 2 HePG2: violin/box plots of execution time for *np*-eSS vs saCeSS2 using 10, 20 and 40 MPI processors. **4**

C Violin/boxplots comparing results in terms of execution time in Azure vs local cluster **5**

1 Introduction

The following figures show details regarding the dispersion of the results discussed in the main text of our article. In each figure, we show (for each method, saCeSS2 and eSS): a green asterisk representing the mean, a typical boxplot figure, and squares in light blue representing the frequency histogram of execution times (i.e. the required time to reach a target value or value-to-reach, VTR). All the experiments were performed using 10, 20 and 40 MPI processors.

A Case study 1 SSP: violin/box plots of execution time for *np*-eSS vs saCeSS2 using 10, 20 and 40 MPI processors.

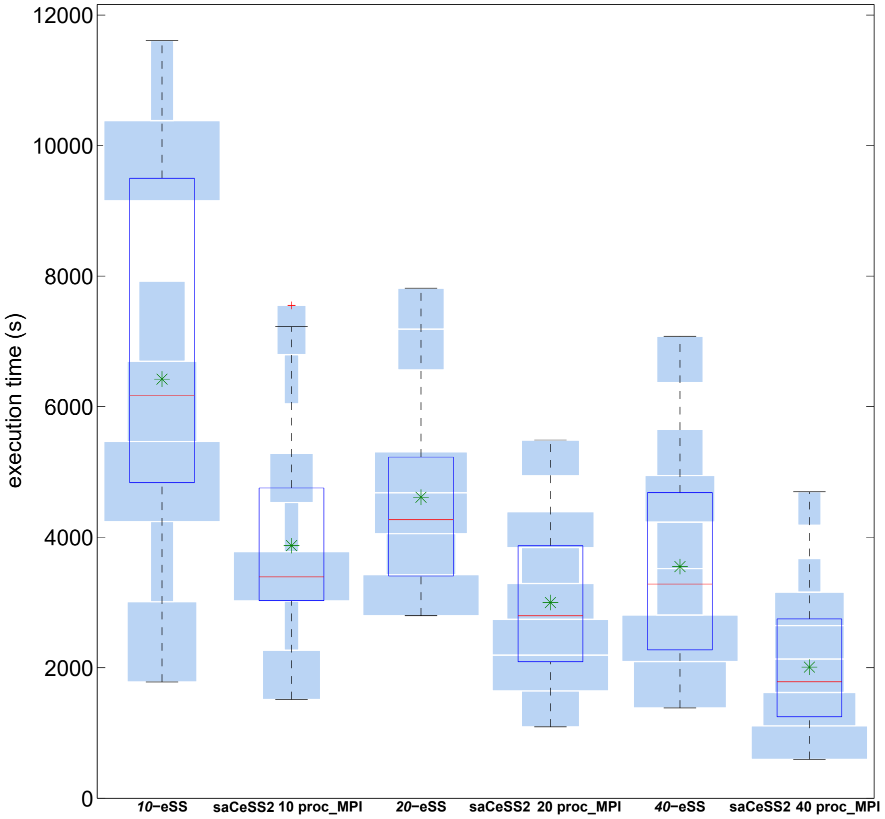

Figure A: Case study 1 SSP: violin/box plots of execution time for *np*-eSS vs saCeSS2 using 10, 20 and 40 MPI processors. VTR=10. Number of independent runs = 20.

B Case study 2 HePG2: violin/box plots of execution time for *np*-eSS vs saCeSS2 using 10, 20 and 40 MPI processors.

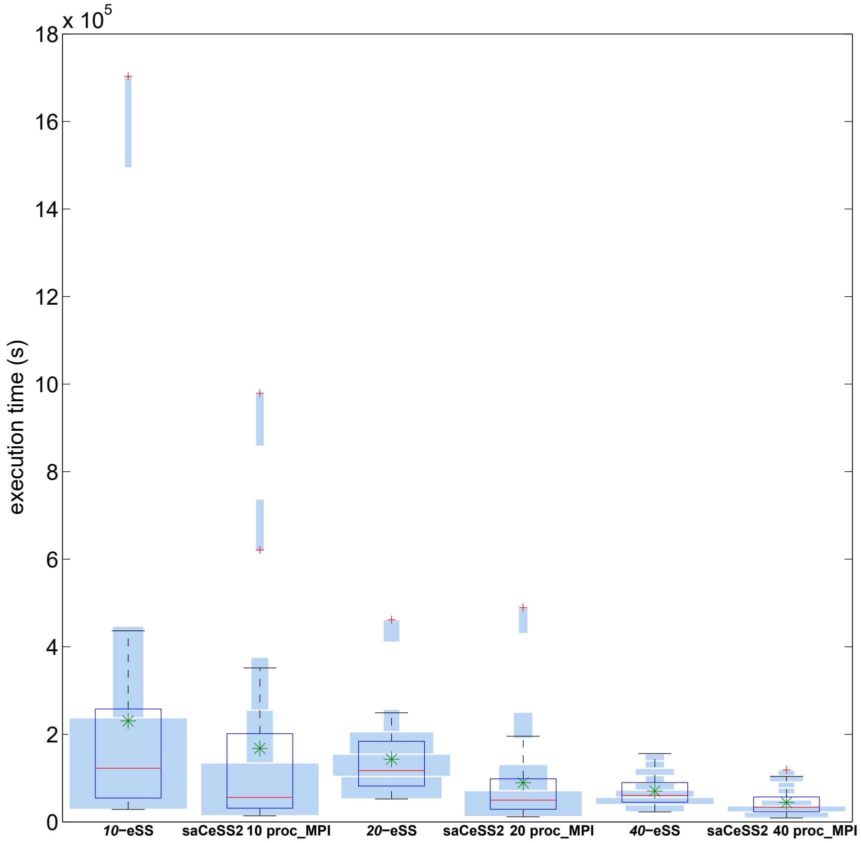

Figure B: Case study 2 HePG2: violin/box plots of execution time for *np*-eSS vs saCeSS2 using 10, 20 and 40 MPI processors. VTR=33. Number of independent runs = 20.

C Violin/boxplots comparing results in terms of execution time in Azure vs local cluster

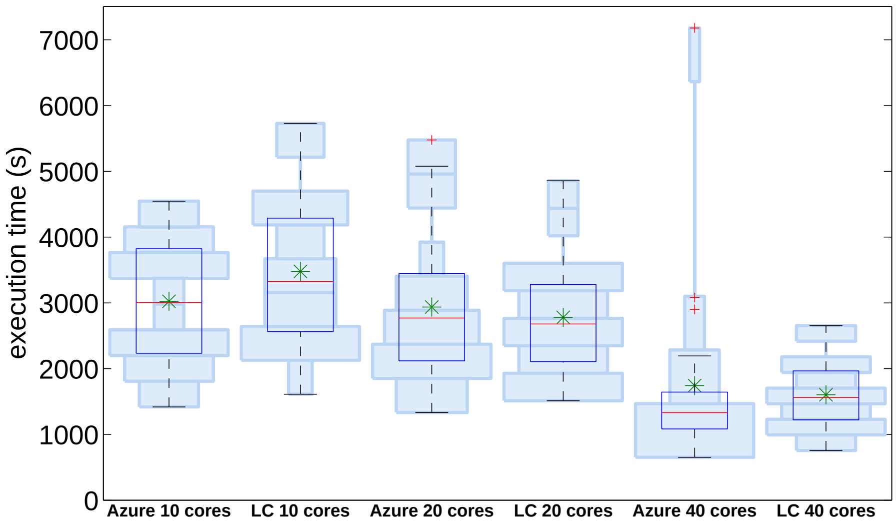

(a) Case study 1: SSP.

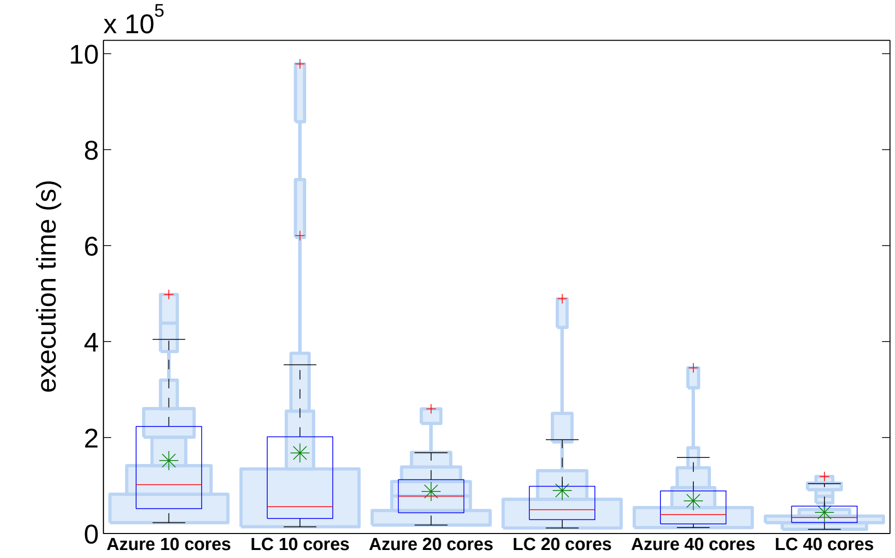

(b) Case study 2: HePG2.

Figure C: Violin/boxplots comparing results in terms of execution time in Azure vs local cluster (LC)
